# Notes and News

**Published:** 1900-06-23

**Authors:** 


### Notes and News.

When Mr. W. Waldorf Astor's eldest son came of age Mr. Astor presented £10,000 to the Maidenhead Cottage Hospital for its permanent endowment.

Professor Wilhelm Röntgen has been awarded the Barnard gold medal of the Colombia University for his *x* ray discoveries.

Lord Lister will deliver the third Harley lecture in connection with the Charing Cross Hospital on October 2nd.

The annual meeting of the Central London Throat and Ear Hospital will be held at the hospital on Tuesday, June 26th, at four p.m.

Plague laboratories are to be established at Freiburg and Heidelberg in Germany, for the purposes of research and diagnoses. They are to be connected with the universities.

The Epidemiological Society of London has reached the fiftieth year of its existence, which event will be celebrated by a commemoration dinner at the Grand Hotel on July 13th.

A society for the study of diseases of children is to be established in London. It is curious that among the many societies which exist on every side, this subject, which is on the one hand so special, and on the other so widely interesting, should have been so long left out in the cold. Mr. Sydney Stephenson, well known in connection with the efforts to control epidemic ophthalmia in our Poor Law schools, is the honorary secretary of the Provisional Committee.

The effort made by the Jenner Institute to avoid being rated for parochial purposes has failed. The case came before the Court of Queen's Bench last week, the institute claiming exemption on the ground that it was a society established exclusively for the purposes of science, and was supported in part by voluntary contributions, and that the making and sale of preventive medicines was a means of disseminating scientific knowledge. It was, however, contended in opposition that the appellants carried on a trade in the production and sale of antitoxins, and ought not to be exempt, and in this view the judges concurred.

A National Congress against Tuberculosis in Portugal is under consideration.

We recently stated that the Needwood Convalescent Home for Sick and Wounded Soldiers at Bournemouth had been closed. The scheme, we are now informed, has been resuscitated, a new committee carrying on the work under the same matron and nursing staff.

The Royal Medical and Chirurgical Society will no longer publish its *Proceedings*, but reports of the discussions on papers communicated to the society will in future appear in its *Transactions*. This will be a great improvement.

The long-talked-of Pasteur Institute for India is now actually about to be established in the hill cantonment of Kasauli, nine miles off the railroad to Simla. The institute will be managed by a committee, and under the medical control of Major Semple, R.A.M.C.

At the last meeting of the Metropolitan Asylums Board the Managers were approached by the Sick Asylum District Board of Poplar and Stepney, with a view to sending a deputation in regard to securing accommodation for children suffering from ophthalmia and ringworm.

The Organising Committee of the Congrès International Electrologie et de Radiologie Medicales have arranged for a considerable reduction of railway fares on French railways for members of the Congress, who should apply to M. Doumer, 57, Rue Nicholas, Leblance, Lille, Belgium, before July 1st.

A children's hospital has been opened at Athens. The scheme was originated by Princess Sophia of Greece, and the whole of the Royal Family of Greece have taken much interest in it. The establishment is arranged in twelve separate buildings, sufferers from various forms of ailment being treated separately. The institution is named the Saint Sophia Hospital.

Lord Dufferin has recently opened a cottage hospital at Dromore, Ireland. Two wards are situated on the ground floor, together with surgery, matron's and committee room, and domestic offices. Four small wards are situated on the first floor. The total accommodation is for fifteen patients. The hospital has been presented to the town by Mr. William Cowan Heron, J.P.

A question having arisen as to the responsibility of the Government for the support and medical treatment of non-commissioned officers and men of the Imperial Yeomanry, City of London Imperial Volunteers and Volunteer companies invalided home from South Africa, who may be desirous of going to their own homes for medical treatment instead of being admitted to military hospitals, it has been decided that those who wish to go home may do so, provided that they are fit to travel, and that they give a written undertaking that they or their friends can guarantee the responsibility for their care and medical attendance, and that they quite understand that no claim for medical attendance will be admitted as a charge against the public. The cost of their conveyance to their homes may, however, be admitted as a public charge.

